# HCG trigger *versus* GnRH agonist trigger in PCOS patients undergoing IVF cycles: frozen embryo transfer outcomes

**DOI:** 10.5935/1518-0557.20200028

**Published:** 2021

**Authors:** Krishna Deepika, Rathore Suvarna, Maria Sumi, Dhoble Snehal, Vohra Arveen, Kamath Anuja, Pranesh Gautham, Rao Kamini

**Affiliations:** 1 Milann, the fertility Centre, Bangalore, India

**Keywords:** GnRHa trigger, hCG trigger, PCOS, FET, live birth rate

## Abstract

**Objective:**

The use of Gonadotrophin releasing hormone agonist (GnRHa), with freeze-all strategy followed by frozen embryo transfer (FET) has been found to eliminate the risk of ovarian hyperstimulation syndrome (OHSS) in women with polycystic ovarian syndrome (PCOS) undergoing IVF cycles. However, physicians still hesitate to routinely use GnRHa as a trigger, replacing human chorionic gonadotrophin (hCG), for concerns of compromised cycle outcome. We aimed to evaluate outcomes following the transfer of embryos in FET cycles obtained from GnRHa trigger in comparison with hCG trigger in PCOS patients of Asian origin.

**Methods:**

Prospective observational cohort study. 210 PCOS patients undergoing IVF in an antagonist protocol who were randomized in the previous study (to evaluate if GnRHa trigger is a better alternative than hCG in PCOS patients to prevent OHSS; Group A: GnRHa trigger (n=92)] and Group B: hCG trigger (n=101)], were followed up in FET cycles to assess the outcomes.

**Results:**

The odds of cumulative live birth rate per stimulation cycle favors GnRHa trigger against the hCG trigger [OR=2.15; (CI 1.2-3.83); *p*=0.008]. A significantly higher number of mature oocytes (19.1±11.7 *versus* 14.1±4.3; *p*<0.001) and blastocysts (4.2±1.63 *versus* 3.26±1.22; *p*<0.001) were available in the GnRHa group as compared to the hCG group.

**Conclusion:**

The cumulative live birth rate was better following transfer of frozen-thawed embryos generated from GnRHa-triggered cycles compared to hCG trigger. Hence, in PCOS undergoing IVF, as a good practice point, hCG trigger should be replaced by a GnRHa trigger with vitrification of all embryos followed by FET.

## INTRODUCTION

There has been an exponential increase in the number of Assisted Reproductive technology (ART) cycles ^(Toner et al., 2016)^, which has led to an increase in the incidence of ovarian hyperstimulation syndrome (OHSS), causing a threat to clinicians ^(Humaidan et al., 2010a)^. In IVF cycles following ovarian stimulation, the mild form of OHSS accounts for 20-33% ^(Humaidan *et al.,* 2013^; ^Yen et al., 1968)^, moderate to severe being 3% to 8%, which increases to 10-20%, in a high risk population of PCOS ^(Delvigne & Rozenberg, 2002^; ^Nastri et al., 2015)^. Moderate and severe OHSS patients are of concern, with attendant morbidity ^(Papanikolaou et al., 2006)^ and mortality in cases of severe OHSS ^(Delvigne & Rozenberg, 2002)^, with one death for every 50,000 treatment cycles as per the World Health Organization report ^(Hugues, 2001^; ^Şükür et al., 2017)^. The culprit for this devastating complication is hCG, “The king”, which has ruled the ART kingdom for more than three decades as ovulation trigger ^(Humaidan & Polyzos, 2014)^. There is worldwide emphasis on the need to eliminate OHSS and one of the most effective strategy for this would be the use of GnRHa, as an alternative trigger for final oocyte maturation. GnRHa has revolutionized ART in the last decade, as it significantly reduces, or nearly eliminates the risk of OHSS ^(Engmann et al., 2006^; ^2008^; ^DiLuigi et al., 2010^; ^Humaidan *et al*., 2010a)^ in women with PCOS undergoing IVF cycles.

However, there still has been reservations concerning the use of GnRHa as a trigger routinely in all PCOS women undergoing IVF cycles for the prevention of OHSS, because of concerns of lower pregnancy rates ^(Humaidan et al., 2005^; ^Kolibianakis et al., 2005)^, with reports on the outcomes, being conflicting. The causes of lower pregnancy rates, could probably, be due to sub-optimal yields of mature oocytes, with few cases of immature oocyte syndrome and empty follicle syndrome (EFS) reported ^(Honnma et al., 2011^; ^Castillo et al., 2012)^, possible adverse effects on oocyte, embryo, endometrium and luteal phase. Although there has been a lot of supporting evidence in the literature to demonstrate that GnRHa trigger hampers embryo implantation due to rapid luteolysis, with associated luteal phase defects ^(Beckers et al., 2003^; ^Kol, 2004^; ^Humaidan *et al*., 2012)^, rather than developmental inability of the oocyte/embryos. Modified luteal support has been considered following embryo transfer in a fresh cycle, which can improve success rates ^(Humaidan *et al*., 2010b^; ^Iliodromiti et al., 2013^; ^Engmann et al., 2006^; ^2008)^, but this may come at a cost of increasing the risk of OHSS ^(Seyhan et al., 2013)^. Therefore, elective cryopreservation of all the embryos followed by a subsequent transfer, presents as a more rational approach in PCOS management. Furthermore, pregnancy rates after frozen embryo transfer following GnRHa has been shown to be comparable with hCG triggered cycles ^(Eldar-Geva et al., 2007^; ^Griesinger et al., 2007^; ^Herrero et al., 2011)^.

Despite this assurance, there still has been reluctance on the part of clinicians, including us, to replace hCG trigger even in indicated cases of PCOS and hyper-responders as a worldwide survey has shown that GnRHa trigger is used only in 5.2% to 36.1% of cases ^(Engmann et al., 2016)^. There is a need for greater clarity on the outcomes of frozen-embryos obtained from GnRHa triggered cycles in comparison with hCG trigger in terms of cumulative probability of achieving a live birth through consecutive transfers of these vitrified-warmed embryos. In addition, the study also intended to evaluate the embryological outcomes in terms of oocyte maturity, developmental and implantation ability of the embryos in both the groups.

## MATERIALS AND METHODS

### Study design and participants

This was a prospective, observational study conducted in a tertiary care center- Milann Fertility Center, Bangalore to assess the frozen-thawed embryo transfer cycle outcome following GnRHa trigger and hCG trigger in PCOS patients. In the previous randomized controlled trial carried out between May 2013 and November 2015 [comparing GnRH agonist with hCG trigger in an antagonist protocol for prevention of OHSS with freeze-all strategy; 210 PCOS patients were randomized; 92 subjects in Group A: GnRHa triggered (n=92) and Group B: hCG triggered (n=101) included for the final analysis ^(Deepika et al., 2016)^], were followed up prospectively over a period of three years. All participants underwent subsequent frozen-thawed embryo transfer cycles, and the treatment outcome of these subjects is reported herein. Approval was obtained from the Institutional Ethical Committee (ECR/773/INST/KA/2012) and the participants signed an informed consent form.

### Patient population

Inclusion criteria: (i) All PCOS [defined as per the ESHRE/ASRM Rotterdam criteria (Rotterdam ESHRE/ASRM-Sponsored PCOS Consensus Workshop Group, 2004) demonstrating two of three criteria:(a) Oligo or anovulation; (b) clinical and/or biochemical signs of hyperandrogenism; (c) polycystic ovaries, defined on ultrasonography as 12 or more follicles measuring 2-9mm in diameter or increased ovarian volume (>10cc)] undergoing first IVF cycle. (ii) Age 20-37 years; (iii) early follicular phase serum FSH concentration (<10.0 IU/l); (iv) body mass index (BMI) >18 and <30 kg/m^2^; (v) presence of both ovaries; (vi) indication for in-vitro-fertilization (IVF)/intracytoplasmic injection (ICSI); (vii) stimulation in GnRH antagonist protocol; (viii) Freeze-all strategy.

Exclusion criteria: (i) Donor cycles using GnRHa trigger; (ii) Patients with hypogonadotropic hypogonadism; (iii) surgical retrieval of sperms.

### Ovarian stimulation

Controlled ovarian stimulation was started on day 2/3 of the cycle, with Recombinant Follicle stimulating hormone (R-FSH), (Gonal-F, Merck Serono) after performing follicle stimulating hormone (FSH), luteinizing hormone (LH), estradiol (E2), progesterone (P4), anti-mullerian hormone (AMH) and a baseline transvaginal scan. The starting dosage was individualized (ranging 112.5 -175IU) and we used a flexible multiple dose antagonist protocol. With three lead follicles ≥ 17mm in diameter, peak E2, LH and P4 concentrations were measured and final oocyte maturation was accomplished with a single dose of 0.2 mg of Triptorelin (Decapeptyl, Ferring), subcutaneously in group A; recombinant hCG (rhCG), (Ovitrelle, Merck Serono) 250mcg subcutaneously in group B. GnRHa was administered at least 12h after the last dose of GnRH antagonist. Transvaginal ultrasound-guided oocyte retrieval was performed 35 hours after the trigger under intra venous sedation with a single lumen oocyte retrieval needle. We used a freeze-all strategy. Post pick-up, we followed all the subjects on days 4 and 7, to assess for OHSS.

### Cryopreservation and thawing

Oocyte maturity was defined as the ratio of mature oocytes (MII oocytes) to the total number of oocytes collected. ICSI was performed in all cases as per the hospital’s standard operating procedure. Fertilization was checked 18h after ICSI, by the appearance of two pronuclei. The embryos were graded as per the Istanbul consensus: Grade 1 (Good): <10% fragmentation, stage-specific cell size and no multinucleation. Grade 2 (Fair): 10-25% fragmentation, stage-specific cell size for majority of cells and no evidence of multinucleation. Grade 3 (Poor): severe fragmentation (>25%), cell-size not stage-specific and evidence of multinucleation. Grade 1 and 2 embryos were taken as top quality embryos and grade 3 embryos were discarded. The blastocysts were graded as: 1-Early; 2-Blastocyst; 3-Expanded; 4-Hatched/hatching; Inner cell mass: 1(Good)- prominent, easily discernible, with many cells that are compacted and tightly adhered together; 2(Fair)- easily discernible, with many cells that are loosely grouped together; 3(Poor)- difficult to discern, with few cells; Trophectoderm: 1(Good)- many cells forming a cohesive epithelium; 2(Fair)-few cells forming a loose epithelium; 3(Poor)- very few cells. As a policy, split freezing was employed when more than 7-8 CG1 embryos were available on day 3, 50% of them were cryopreserved in the cleavage stage and the remaining were cultured to blastocyst and then frozen. The embryos were then vitrified by open system using cyro-lock with 15% ethylene glycol, 15% dimethylsulphoxide (DMSO) and 0.5mol/L sucrose as cryoprotectants (Sage vitrification kit, Origio). For blastocysts, the blastocele was collapsed using laser (Octax, MTG) and then vitrified using the same protocol as described for day-3 embryos. The embryos selected for transfer were thawed on the day of transfer using 1.0M sucrose (Sage thawing kit, Origio). Following thawing, we assessed the embryo quality by morphologic evaluation. For cleavage embryos, blastomere survival of ≥ 50% (with clear cellular boundaries and no degeneration); and for blastocyst, the ability of the blastocele to re-expand within 2-6 h post thaw was identified as a viable embryo. If not, the embryos were taken as failure to survive and were discarded (Consensus, Istanbul). The survival rate of the embryos following thawing was calculated as the number of viable embryos to the number of those thawed.

### Frozen embryo transfer

All FET cycles were performed in a hormone replacement cycle with a 6mg daily dose of orally administered estradiol (Progynova; Zydus Cadila, German Remedies). Patients were to start FET cycle within 3 months after oocyte pick-up. When the endometrium evaluated by transvaginal sonography (TVS) was >8mm with triple layer morphology, it was considered mature. This was followed by endometrial priming with 3 days of injectable progesterone (gestone 50mg; Ferring) for cleavage embryos and 5 days for blastocysts. If the endometrial thickness was <7 mm on day 9, Oestrogel (Besins, Belgium) was added and the dose of estradiol was increased to 12mg. If the endometrial thickness remained less than 7 mm, in spite of prolonged estradiol priming, the cycle was cancelled. The maximum number of embryos thawed and transferred per FET cycle was three in cleavage embryos and two in blastocyst. The day of embryo transfer (D3 or D5) was a clinical decision made depending on the endometrial thickness and the availability of good-quality embryos. Embryo transfer (ET) was performed under ultrasound guidance using Cooks catheter (K-JETS-7017-SIVF, Cook Medical, Syndey IVF). Luteal phase supplementation was administered for 14 days with vaginal progesterone and estradiol, and continued until 10 weeks of gestation, when clinical pregnancy (CP) was achieved. The CP rate was calculated as the number of cases with evidence of at least one gestational sac by TVS, divided by the number of transfers. The implantation rate was expressed as the number of gestational sacs seen on TVS to the number of embryos transferred. Miscarriage was defined as first-trimester pregnancy loss occurring after the first documentation of pregnancy on ultrasound from the fifth to the sixth week of gestation, until the 12^th^ week, and miscarriage rate was defined as the number of miscarriage divided by the number of transfers. Live birth was defined as birth beyond the period of viability (28 weeks of gestation) and the live birth rate was the ratio of live births to embryo transfers. Every woman was followed up until the first live birth or until three transfers if sufficient embryos were available. The cumulative live birth rate was defined as the number of live births per patient after three frozen embryo transfer cycles or exhaustion of all available embryos before three embryo transfer cycles per stimulation cycle. FET attempts for a second live birth from the same initial treatment cycle were excluded.

### Outcome measures

Primary outcome: Cumulative live birth rate.

Secondary outcomes: MII rate of oocytes, availability of top quality embryos on day 3 (Grade 1 and grade 2) and blastocysts on day 5, OHSS rate, survival rate of cryopreserved embryos, implantation rate, clinical pregnancy rate, miscarriage rate and multiple birth rate.

### Statistical analysis

We analyzed the data using the Statistical Package for Social Sciences, version 16.0 (SPSS, USA). The continuous variables were expressed as mean ± SD, and the categorical values expressed as percentages were analyzed using the chi square test. Independent sample t-test was used for continuous variables, which had a normal distribution. We used the odds ratio to evaluate the association of outcomes across the groups. A *p* value <0.05 was considered statistically significant.

## RESULTS

### Baseline and stimulation cycle characteristics

The baseline and stimulation characteristics were similar in both groups (Table 1). However, the number of dominant follicles ≥17mm, the number of intermediate follicles between 14 to 16mm and peak E2 levels on trigger day was found to be significantly higher in group A, as compared to group B (Table 1).

**Table 1:** Baseline and stimulation characteristics

Variables	Group A (GnRHa) (n=92)	Group B (hCG) (n=101)	*p*
Age (years)	29.1±3.8	29.06±3.6	0.940
Primary infertility *n* (%)	54 (58.7%)	64 (63.3%)	0.513
Secondary infertility *n* (%)	38 (41.3%)	37 (36.7% )	0.51
Duration of infertility	6.8±2.8	6.2±2.3	0.168
Irregular menstrual cycles *n* (%)	45 (48.9%)	51 (50.5%)	-
Clinical Hyper-androgenemia *n* (%)	31 (33.7%)	36 (35.6%)	-
BMI (kg/m^2)^	25±3.7	24.9±3.8	0.901
Day 2 FSH	5.2±1.5	5.1±1.3	0.683
AFC^¥^	26.3±4.8	25.1±4.5	0.074
AMH^£^(ng/ml)	5.7±2.8	5.9±2.6	0.561
Dosage of gonadotrophin	1845±707	2095±906	0.127
Duration of stimulation	10±1.2	10±1.4	0.876
DF^ǂ^≥17	12.7±4.3	10.9±2.9	0.001
IMF^Ῡ^14-16mm	11.7±3.9	9.9±3.3	0.001
Peak Estradiol	4678.1±1331	3870.4±1556	0.001
Peak Progesterone	1.76±1.1	1.39±1.1	0.052

*p*<0.05 = statistically significant. AFC^¥^-Antral follicle count; AMH^£^-Anti-mullerian hormone; DF^ǂ^-Dominant follicle; IMF^Ῡ^-Intermediate follicle

### Embryological and cycle outcomes

Table 2 summarizes the embryological and cycle outcomes in both groups. Although the blastocyst conversion was similar in both groups, a significantly higher number of blastocysts was available in group A. The incidence of moderate to severe OHSS in the hCG group was 37.6% and 0% in GnRHa group (*p*<0.001).

**Table 2:** Embryological and cycle outcomes

Variables	Group A (GnRHa) (n=92)	Group B (hCG) (n=101)	*p*
Number of oocytes	23.5±7.8	20.8±5.4	0.006
Mature oocytes (MII)	19.1±11.7	14.1±4.3	<0.001
Fertilized oocytes (2PN)	15.6±5.6	11.7±3.6	<0.001
Top quality cleavage embryos	12.9±3.32	9.09±2.99	<0.001
Blastocysts	4.2±1.63	3.26±1.22	<0.001
Blastocyst conversion	59.9%	58.2%	0.689
OHSS *n* (%)	1 (0.52%)	91 (47.4%)	<0.001

Values are expressed as mean± SD. *p*<0.05= statistically significant.

### Clinical outcomes-FET

**Figure 1:**
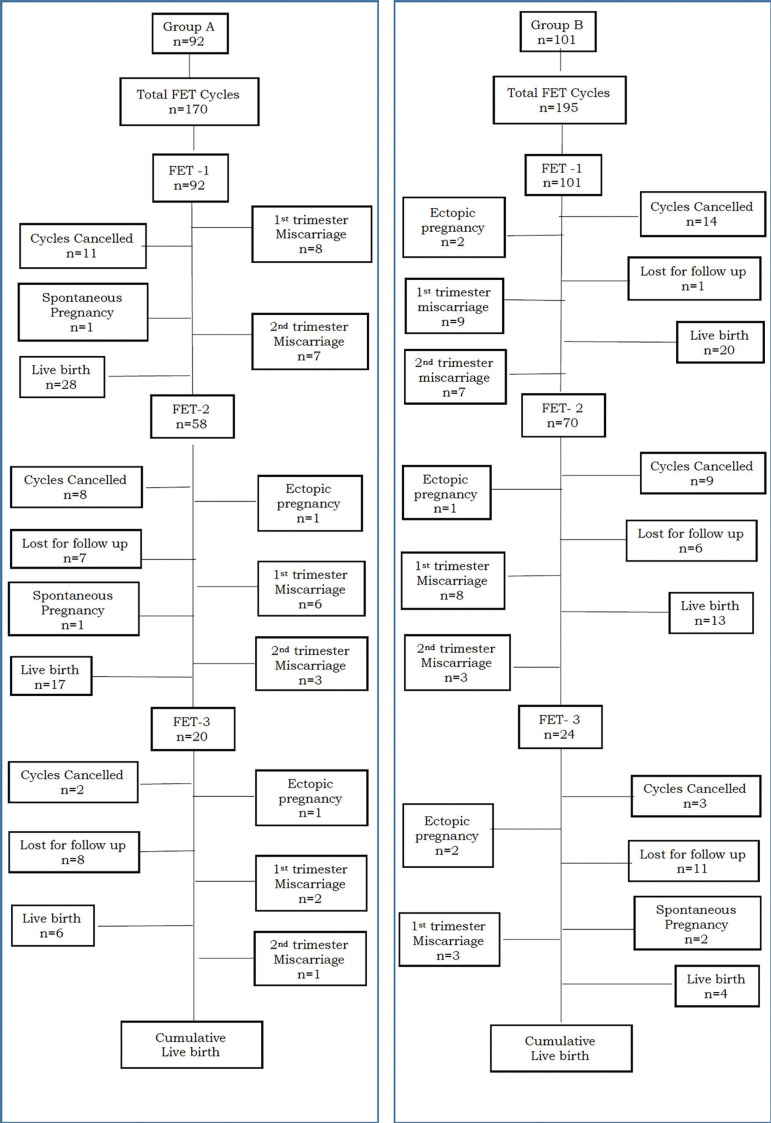
Subject flow chart

Subjects entering the first FET were 92 in group A, and 101 in group B, with a total of 365 FET cycles carried out across the groups, (170 cycles in GnRHa and 195 cycles in hCG group) [Figure 1. Subject Flow chart]. In total, we cancelled about 21 cycles (12.3%) in group A and 26 in group B (13.3%). The reasons for cycle cancellation were suboptimal endometrium, fluid in the endometrial cavity, premature rise of progesterone were not statistically significant between the two groups. However, cycle cancellation due to poor quality embryos following thawing was significantly higher in the hCG group than in the GnRHa group [(9/195=4.6%) *versus* (2/170=1.2%); (*p*=0.056)], respectively. Individuals entering 2^nd^ and subsequently 3^rd^ FET were either those who had failed to achieve a live birth with surplus embryos frozen or those in whom the cycles were cancelled for various above-mentioned reasons. Accordingly, 193 patients underwent the first FET cycle, 128 patients underwent a second FET and 44 patients underwent a third FET cycle across both the groups. Of reference, patients following an FET cycle, who had failed to achieve a live birth, with frozen embryos, yet did not return for transfer was similar in both groups (15/92=16.3% *versus* 18/101=17.8% *p*=0.78). Almost all subjects following oocyte retrieval returned for their 1^st^ FET, except for one in group B, with a significant proportion being lost following a 2^nd^ FET (n=13) and a 3^rd^ FET (n=19) across the groups. Following failed FET cycles, four individuals (4/193=2%) achieved spontaneous pregnancy. Table 3 summarizes the clinical outcomes of FET cycles.

**Table 3:** Clinical outcome-FET cycles

Variables	Group A (GnRHa) (n=92)	Group B (hCG) (n=101)	*p-*value
FET cycles attempted (365)	170	195	*-*
FET cycles cancelled *n*(%)	(21/170) 12.3	(26/195) 13.3	0.776
FET cycles with cleavage/blastocyst transfers a. cleavage *n* (%) b. blastocyst *n* (%)	(46/149) 30.8 (103/149) 69.1	(75/169) 44.3 (94/169) 55.6	0.013 0.013
Mean number of embryos thawed a. cleavage b. blastocyst	3.02±0.57 2.15±0.53	3.19±0.48 2.16±0.40	0.079 0.881
Survival rate *n* (%) a. cleavage *n* (%) b. blastocyst *n* (%)	(123/142) 86.6 (198/221) 89.5	(209/239) 87.4 (181/203) 93.3	0.815 0.894
Mean number of embryos transferred a. cleavage b. blastocyst	2.62±0.61 1.92±0.3	2.79±0.41 1.93±0.26	0.068 0.803
Clinical pregnancy/ET *n* (%)	(79/149) 53	(70/169) 41.4	0.039
Implantation rate *n* (%)	(94/321) 29.2	(82/390) 21	0.011
Miscarriage rate *n* (%)	(16/149) 10.73	(20/169) 11.83	0.757
Live birth per ET *n* (%)	(51/149) 34.2	(37/169) 21.89	0.014
Singleton births *n* (%)	(38/79) 48.1	(25/70) 35.7	0.127
Multiple births *n* (%)	(13/79) 16.4	(12/70) 17.1	0.909
Cumulative live birth rate *n* (%)	(51/92) 55.43	(37/101) 36.63	0.009

expressed as number (percentage). *p*<0.05= statistically significant

The mean endometrial thickness was comparable between the groups (9.5±1.75 *versus* 9.8±1.35mm, *p*=0.07). The number of cleavage embryos and blastocysts thawed as well as transferred per cycle was comparable in both the groups (Table 3). Accordingly, 711 embryos were transferred, with 379 blastocysts and 332 cleavage embryos replaced in 318 cycles across the groups. The odds for overall survivability of embryos (cleavage + blastocyst) across the groups was 1.01 [(CI-0.66-1.57); *p*=0.92]. The CP rate following 1^st^ FET (43/81=53% *versus* 36/87=41.4%; *p*=0.13); following 2^nd^ FET (27/50=54% *versus* 25/61=41%; *p*=0.35); after 3^rd^ FET (9/18=50% *versus* 9/21=42.8%; *p*=0.77), in group A and B, respectively. The odds of CP per ET was significantly higher in group A [OR=1.59; CI (1.02-2.48); *p*=0.04]. The odds of cumulative CP per patient per stimulation cycle was significantly higher in group A [OR=2.69; CI (1.3-5.54); *p*=0.006]. The odds of implantation was found to favor the GnRHa group [OR=1.56; CI (1.1-2.91); *p*=0.011]. There were seven ectopic pregnancies (7/318=2.2% per cycle started). The singleton live birth rate was 42.3% and that for multiple births was 16.8% (16.1% twins, 0.6% triplets) across the groups (Table 3).

The live birth rate per first FET cycle was 34.6% and 22.9% [OR=1.77; (CI 0.89-3.48); *p*=0.097] in the GnRHa and the hCG group, respectively, with no significant differences when assessed across individual FET cycles (cycles 2 & 3). The odds of cumulative live birth rate per patient starting a frozen embryo transfer cycle was found to favor the GnRHa trigger as compared with the hCG trigger [OR=2.15; (CI 1.2-3.83); *p*=0.008].

## DISCUSSION

HCG has lucratively been used as a surrogate for the endogenous mid-cycle luteinizing hormone (LH) surge in all IVF cycles for follicular maturation, because of its similarities with LH ^(Ascoli et al., 2002)^. However, the prolonged luteotropic action of hCG ^(Yen et al., 1968)^ increases the risk of OHSS in PCOS and in hyper-responders. GnRHa has emerged as an alternative trigger, due to its initial flare effect, releasing endogenous gonadotrophins from the pituitary. However, due to its considerably short-lasting LH surge (24-36 h), it causes rapid luteolysis ^(Humaidan et al., 2005^; ^Kolibianakis et al., 2005)^, with a drastic fall in steroid hormones and vascular endothelial growth factor (VEGF), the cytokine primarily responsible for OHSS ^(Cerrillo et al., 2009)^. A beneficial result of this is that it reduces the incidence of OHSS, but the fear has been with compromised cycle outcomes and reduced pregnancy rates.

The compromised cycle outcome and reduced pregnancy following GnRHa trigger was attributed to the probable ill effects of GnRHa on the quality of oocyte, embryo and implantation potential of the embryo. Initial RCTs which compared the clinical outcomes of GnRHa and hCG triggers in normoresponders undergoing IVF ^(Fauser et al., 2002^; ^Kolibianakis et al., 2005^; ^Humaidan et al., 2005)^, reported significantly lower pregnancy rates and higher early pregnancy losses in the GnRHa group. With these disappointing outcomes, the studies were prematurely stopped ^(Kolibianakis *et al.,* 2005^; ^Humaidan *et al.,* 2005)^. Further, a meta-analysis by ^Griesinger et al. (2006)^ reported that GnRH agonist trigger is associated with a significantly reduced likelihood of achieving a clinical pregnancy (0.21, 0.05-0.84; *p*=0.03), with a pregnancy rate of 7.9% and 29.9% per randomized patient and an early pregnancy loss rate of 67.6% and 12.7% in the GnRHa and hCG triggered groups, respectively. These worrying rates were postulated to be due to either poor oocyte/embryo quality or luteal phase deficiency. Later, it was demonstrated that the proportion of mature oocytes, fertilized oocytes, and embryos obtained from donors triggered with GnRHa was similar to that of hCG trigger ^(Shapiro et al., 2007^; ^Erb et al., 2010)^. These embryos, when transferred to oocyte recipients, resulted in good implantation and pregnancy rates ^(Acevedo et al., 2006^; ^Sismanoglu et al., 2009^; ^Bodri et al., 2009^; ^Melo et al., 2009)^, thereby discounting any detrimental effect of GnRHa on oocyte/embryo quality. The lower live birth rates ^(Humaidan *et al.,* 2005^; ^Kolibianakis *et al*., 2005)^ was found to be due to defective luteal phase as significantly lower levels of E2, P4, inhibin A, and inhibin pro-aC levels were observed, impairing the endometrial receptivity and implantation ^(Fauser *et al.,* 2002^; ^Humaidan *et al.,* 2005^; ^Nevo et al., 2003)^. Further, to substantiate the compromised outcome following GnRHa is more due to luteal phase defects, than due to ill-effects on the oocyte or embryo, it has been established that the clinical outcome following transfer of frozen-thawed embryos obtained in GnRHa triggered cycles was the same as in those cycles triggered with hCG ^(Eldar-Geva et al., 2007^; ^Griesinger *et al.,* 2007^; ^Herrero et al., 2011)^. Although, these data provide convincing evidence, there is still some apprehensions concerning the use of GnRHa trigger, in terms of oocyte quality, embryo quality and live birth rates. Thus, our study intended to assess the live birth rate in a freeze-all strategy in PCOS, following the transfer of embryos triggered with GnRHa in comparison to those from hCG triggered cycles.

Our study found a statistically significant higher clinical pregnancy rate following the transfer of embryos obtained from the GnRHa group as compared with that from the hCG group in frozen-thawed cycles (Table 3). This could be associated with a higher number of oocytes retrieved, greater maturity, better fertilization, greater number of top quality embryos and a higher number of blastocysts obtained in the GnRHa group in comparison to the hCG group. A higher number of follicles was observed in the GnRHa group, accounting for the retrieval of a higher number of oocytes reaching statistical significance. This difference in oocyte numbers may be a reflection of different responses from each individual between the two groups, which probably is a chance finding. Importantly, we witnessed an increase in MII oocytes (average of 5) retrieved in the GnRHa triggered group, adding to the impact on the clinical outcome. This could possibly, be the result of a physiological FSH surge, which induces the formation of LH receptors on the luteinizing granulosa cells, promoting nuclear maturation and cumulus expansion ^(Eppig, 1979^; ^Humaidan et al., 2005^; ^2010b)^. The availability of top-quality cleavage embryos on day 3 was significantly higher in the GnRHa group (91.3%) than the hCG group (74.3%). In concordance, few trials have reported that the use of an agonist trigger produced comparable or slightly superior embryo quality compared with hCG trigger ^(Humaidan *et al.,* 2009^; ^2010b^; ^2011^; ^DiLuigi et al., 2010)^. The same has been substantiated in donor cycles, reporting a higher yield of good quality embryos in the GnRHa triggered group ^(Melo et al., 2009^; ^Acevedo et al., 2006^; ^Erb et al., 2010)^.

A better embryological outcome observed in the GnRHa group compared with the hCG group (Table 2 and 3), could be associated with the differences in the duration, profile and physiological events following the two triggers ^(Yding Andersen et al., 1993)^. LH and hCG differ in structural features, such as the presence of a carboxyl terminal peptide, the type and amount of glycosylation. The half-life of LH is shorter (60-120min), whilst the half-life of hCG exceeds 24h, exerting a higher biological activity. Because of these differences, although both LH and hCG act on the same receptor, luteinizing hormone-chorionic gonadotropin receptor (LHCGR), there is preferential activation of different signal transduction pathways and, eventually, different cell responses ^(Ulloa-Aguirre et al., 2011)^. LH is more active than hCG on pAKT and extracellular signal regulated kinase (ERK1/2) phosphorylation, causing granulosa cell proliferation and differentiation ^(Craig et al., 2007)^. Whilst, hCG is more active than LH in activation of cyclic AMP-protein kinase A (cAMP/PKA), steroidogenesis and potentially pro-apoptotic pathways ^(Casarini et al., 2012)^. HCG generates more intracellular cAMP accumulation and increases progesterone concentrations within the follicular fluid ^(Yding Andersen *et al.,* 1993)^.

The longer hCG half-life and increased follicular fluid progesterone levels causes over-luteinization of the recruited follicles ^(Erb et al., 2010)^, affecting oocyte and embryo quality which is reflected by the finding of a higher number of poor quality embryos in the hCG group 26 (25.7%), as compared to the GnRHa group 8 (8.7%); (*p*=0.002). On the contrary, a higher number of top quality embryos on day 3 was seen in the GnRHa group, yielding a significantly higher number of blastocysts (Table 2). As a result, the GnRHa group had a statistically higher number of FET cycles with blastocyst transfer (69.1% *versus* 55.6%; *p*=0.014) than the hCG group. ^Gurbuz et al. (2016)^ showed that wherein time-lapse imaging of the embryos obtained from GnRHa, cleaved faster than the embryos obtained from hCG triggered cycles in antagonist protocols. It has been well established that early cleavage embryos results in a higher fraction of good-quality embryos and blastocysts ^(Cruz et al., 2012^; ^Wong et al., 2010)^. Probably, a time-lapse imaging would have enabled us to compare the exact morphokinetics of the embryos in both groups; it was not done in our study, due to non-availability of this facility.

The live birth rate in women at risk of OHSS triggered with GnRHa, where fresh transfer was carried out, intensifying luteal support was found to be comparable with that of frozen transfer, respectively [27.1% and 20%; *p*=0.4, RR=1.36 (0.65-2.81)] ^(Imbar et al., 2012)^. However, rescuing luteal phase with a bolus of hCG in women at risk of OHSS has resulted in a few cases of severe OHSS [2/ 275 (0.72%) ^(Iliodromiti et al., 2013^; 6/23 (26%) ^Seyhan et al., 2013)^]. Although, the luteal phase can be rescued to enable fresh ET following GnRHa trigger, the concerns, being, late onset OHSS, which tends to be more severe, compromised endometrial receptivity and an ideal (yet to be defined) luteal phase support. Additionally, vitrification has provided us exceptionally good survivability of embryos following thawing. Further, a meta-analysis by ^Roque et al. (2013)^ proved a significantly higher implantation and CP rates, a significantly higher ongoing pregnancy rate ^(Balaban et al., 2008)^, better obstetric and perinatal outcomes ^(Maheshwari et al., 2012)^ in FET cycles when compared to fresh ETs. Hence, we preferred to avoid fresh transfers, freeze all embryos and transfer them subsequently, the so-called segmentation strategy ^(Devroey et al., 2011^; ^Garcia-Velasco, 2012)^. However, cycle segmentation might not be acceptable for all due to ethical, legal or social reasons, the addition costs involved in freezing and the risks to embryo viability during the freezing and thawing processes. Moreover, cryopreservation may produce alterations in the embryonic genome integrity, which are undetectable by traditional assays. Such modifications might have long-term implications of epigenetic disorders in children born from these vitrified embryos ^(Kopeika et al., 2015)^, with limited number of long-term follow-up studies until now.

Following thawing, the survival rate of vitrified embryos and the morphologic quality of vital embryos that were transferred were found to be similar in both the groups. Further, the survival rate of vitrified cleavage embryos in the GnRHa group (86.6%), is within the range of the survival rates following hCG trigger, as reported by few other authors, as 70% to 95% ^(Balaban et al., 2008^; ^Rezazadeh Valojerdi et al., 2009)^. Additionally, the survivability of vitrified blastocysts following GnRHa (89.5%), is similar to that following hCG trigger as reported in two large studies, as 77% ^(Van Landuyt et al., 2011)^ and 85.7% ^(Takahashi et al., 2005)^. Further, the GnRHa group witnessed a higher implantation rate per FET cycle when compared to the hCG group (Table 3). The findings concur with the previous morphokinetics-based studies, wherein, it has been explicated that the proportion of competent embryos with higher implantation potential was significantly higher in the GnRHa group when compared with the hCG-triggered group ^(Fenwick et al., 2002^; ^Lemmen et al., 2008^; ^Wong et al., 2010)^.

**Figure 2:**
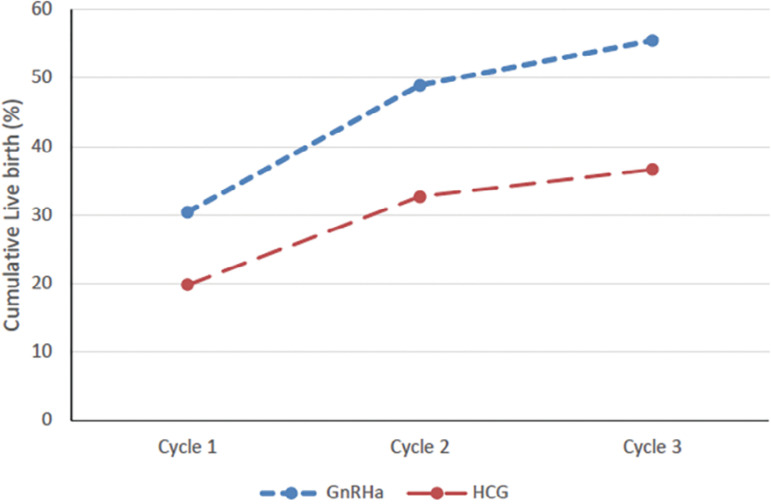
Cumulative Live birth rate per patient in FET cycles following GnRHa trigger (blue dotted line) and hCG trigger (red dotted line).

Women undergoing up to three FET attempts were considered for the analysis, as the efficacy of further attempts is questionable, bearing in mind, the fact that good quality embryos would be transferred in the first few cycles. Thus, the highest CP rates were seen in the first and second cycles compared with the third one, establishing that patients with relatively good prognosis were more likely to achieve a live birth. Following three consecutive frozen transfers, remarkably, the cumulative live birth rate in the GnRHa group was 55.4% *versus* 36.6% in hCG group, and a trend of higher birth rates was consistently found in the GnRHa group (Figure 2). Thus, our study derives a meaningful aspect that about 55% of infertile PCOS patients achieved a live birth with one ovarian stimulation cycle following GnRHa trigger. Further, after three failed FET attempts, it could be that these patients could decrease their time to pregnancy either by considering pre-implantation screening of the remaining frozen embryos as a selection tool, or by starting a fresh ovarian stimulation cycle. It is noteworthy that a significant proportion of cumulative take-home babies came from the transfer of vitrified blastocysts (60.7% and 62.1%) across the groups. The cumulative live birth rate was higher with the transfer of blastocysts (61.3%) than cleavaged embryos (38.6%), in both groups and thus, a live birth can be achieved earlier with blastocyst than with early stage embryo transfer.

The Live birth rate per FET cycle was 34.2% and 21.9% [OR=1.86 (CI 1.13-3.05); *p*=0.015] in the GnRHa and the hCG group, respectively. A significant difference in the live birth rate per FET cycle was in favor of the GnRHa trigger, of 12.3%. The live birth rate per FET reported in a large retrospective cohort study of 5,85,065 ART treatment cycles performed between 2002 and 2013 using the Australian and New Zealand Assisted Reproduction Database (ANZARD) was 23.3%, the adjusted odds resulting in a live birth was 0.86 (95% CI 0.82-0.90) ^(Chambers et al., 2016)^. In the Canadian ART Registry (CARTR), the take-home rate in 3,224 FET cycles in a non-selected patient population was 17.8% ^(Gunby *et al*., 2011)^. As per the HFEA report, the live birth rate per started FET cycle was 24.8% in 2013 ^(Human Fertilisation and Embryology Authority (HFEA), 2016)^. The live birth rates in FET cycles obtained from embryos following GnRHa trigger presented in this study are well above the range of that following the hCG trigger, as reported in the various large-scale studies mentioned above. The favorable outcome associated with the FET cycles following GnRHa trigger is the result of better oocyte maturity and better quality embryos, with developmental potential. We thus uphold the recommendation that a time has come for a paradigm shift in triggering policy, replacing hCG, endorsing the statement by Humaidan, “The King is dead, long live the King” ^(Humaidan & Alsbjerg, 2014^; ^Humaidan & Polyzos, 2014)^.

The strengths of the study being, homogenous select population of PCOS, comparing the outcome of FETs following GnRHa and hCG trigger in a freeze-all cycle, without any negative influence of fresh transfer, avoiding confounding biases. The power of randomization present in the initial study ^(Deepika et al., 2016)^ has still been preserved in this observational study, because the same set of subjects were followed up as FET cycle without any impact on the baseline risk for accomplishment of live births. The study derives more precise estimates of the outcomes of interest, the cumulative live birth rates and embryological outcomes in both the groups. Limitations of the study being, the population included moderately younger age (average 29.1±3.1), non-obese, PCOS undergoing the first IVF cycle with substantially good prognosis. Although a good number of top quality embryos were available on day 3, all of them could have been cultured to blastocyst and then frozen, instead of split freezing which could have minimized bias. Because of this, the study included both cleavage and blastocyst transfers across the groups. However, this lack of homogeneity is less likely to significantly affect our findings. The obstetric, neonatal and importantly, the long-term outcomes of children will be of immense value, a subsequent analysis being planned in the near future.

In conclusion, the transfer of frozen-thawed embryos obtained from GnRHa-triggered cycles in PCOS resulted in higher cumulative live birth rate compared with the hCG trigger. Triggering with GnRHa, yielded more mature oocytes and better quality embryos with a higher developmental potential. Adopting GnRHa as a routine trigger policy in PCOS, is physician and patient friendly, as it provides a better cycle outcome, almost abolishing the risk of OHSS.
